# Dynamic Shape Transformation of a DNA Scaffold Applied for an Enzyme Nanocarrier

**DOI:** 10.3389/fchem.2021.697857

**Published:** 2021-06-24

**Authors:** Peng Lin, Huyen Dinh, Eiji Nakata, Takashi Morii

**Affiliations:** Institute of Advanced Energy, Kyoto University, Kyoto, Japan

**Keywords:** DNA origami, dynamic shape transformation, fluorescence resonance energy transfer, enzyme, nanocarrier

## Abstract

Structural programmability and accurate addressability of DNA nanostructures are ideal characteristics for the platform of arranging enzymes with the nanoscale precision. In this study, a three-dimensional DNA scaffold was designed to enable a dynamic shape transition from an open plate-like structure to its closed state of a hexagonal prism structure. The two domains in the open state were folded together to transform into the closed state by hybridization of complementary short DNA closing keys at both of the facing edges in over 90% yield. The shape transformation of the DNA scaffold was extensively studied by means of the fluorescence energy transfer measurement, atomic force microscope images, and agarose gel electrophoretic analyses. A dimeric enzyme xylitol dehydrogenase was assembled on the DNA scaffold in its open state in a high-loading yield. The enzyme loaded on the scaffold was subsequently transformed to its closed state by the addition of short DNA closing keys. The enzyme encapsulated in the closed state displayed comparable activity to that in the open state, ensuring that the catalytic activity of the enzyme was well maintained in the DNA nanocarrier. The nanocarrier with efficient encapsulation ability is potentially applicable for drug delivery, biosensing, biocatalytic, and diagnostic tools.

## Introduction

Enzymes are spatially organized in the cell to implement specific sequential reactions in the compartments, such as membrane-bound organelles, bacterial microcompartments, and multienzyme complexes (Agapakis et al., 2012). The organization of such enzyme complexes often relies on the specific scaffolds of proteins or the membrane to achieve the high efficiency and the specificity of enzymatic reactions (Conrado et al., 2008; Küchler et al., 2016). Typical examples are found in the following enzymes: ribulose 1,5-bisphosphate carboxylase/oxygenase (RuBisCO) and carbonic anhydrase (CA) packed in carboxysome (Bonacci et al., 2012), plant cytochrome P450 enzymes on the endoplasmic reticulum (ER) membrane (Gou et al., 2018), and electron transport complexes arranged on the cyanobacterial thylakoid membrane (Liu 2016). In these compartments, the reactants in low concentrations are believed to be effectively transferred through spatially arranged enzymes, thereby channeling metabolites to drive favorable reactions and preventing the toxic side reactions by intermediates.

Inspired by the nature systems, individual- or multienzyme complexes have been encapsulated into a wide range of materials, such as proteins (Brasch et al., 2017), lipid vesicles (Walde and Ichikawa 2001), and polymers (Klermund et al., 2017). However, applications of these carriers were limited due to the low-enzyme loading yields and the difficulty in controlling the accurate locations and stoichiometry of enzymes. These obstacles were tackled by DNA nanotechnology. A typical example of DNA nanostructures, DNA origami (Rothemund 2006; Douglas et al., 2009), folds a long single-stranded DNA into predesigned addressable 2D and 3D DNA structures through the hybridization of appropriate staple strands and provides ideal platforms for the assembly of various functional macromolecules (Hong et al., 2017). Besides the static DNA structures, dynamic DNA structures induced by the hybridization of short DNA (Simmel et al., 2019), aptamer switches (Rangel et al., 2020), temperature (Turek et al., 2018), or pH (Kim et al., 2017) changes were constructed to exhibit controlled translational or rotational movement, providing a great potential for applications in drug delivery, biosensing, and biocatalysis (DeLuca et al., 2020). Douglas et al. constructed an aptamer-gated DNA robot in hexagonal barrel, which was opened by antigen proteins like platelet-derived growth factor (PDGF) and transported antibody to the targeting cells (Douglas et al., 2012). Li et al. incorporated the nucleolin-binding aptamer to a DNA origami tube that carried thrombin. Nucleolin expressed by tumor-associated endothelials acted as a trigger of the aptamer locks and opened the tube, which subsequently exposed thrombin in the blood to result in tumor necrosis (Li et al., 2018). Grossi et al. built a DNA nanovault with reversible opening/closing process induced by DNA strand displacements. It was demonstrated that closing the vault significantly reduced the enzyme activity of alpha-chymotrypsin (α-Ct) encapsulated in the vault (Grossi et al., 2017). Xin et al. regulated the cascade reaction of glucose oxidase (GOx) and horse radish peroxidase (HRP) by a DNA tweezer, which could switch between open and closed states driven by a DNA strand displacement reaction. The observed higher overall enzyme reaction efficiency in the closed state was attributed to the shorter distance of two enzyme-modified DNA arms (Xin et al., 2013).

The dynamic environmental changes for enzymes in the cell could be mimicked by changing the environment of enzymes through the dynamic reconfiguration of DNA devices by altering the solution temperature (Juul et al., 2013), pH (Ijäs et al., 2019), or ion concentrations (Marras et al., 2018). However, the variation of these factors at the same time modulates the enzyme activity and affects the stability of DNA origami. Juul et al. introduced a temperature-controlled system to encapsulate or release the enzyme HRP using a preassembled and covalently closed 3D DNA cage structure. The system allowed the entrance or release of HRP at 37°C, with residing HRP in the central cavity of the cage at 4°C (Juul et al., 2013). Ijäs et al. constructed a solution pH-responsive DNA origami nanocapsule that can be loaded with HRP, and reversibly opened and closed by changing the solution pH from 6.4 to 7.8 (Ijäs et al., 2019). Such dynamic DNA devices driven by tuning the solution temperature or pH would only be applicable to the enzyme with high stability, such as HRP. The dynamic shape transformation triggered by the DNA hybridization, or the DNA fuels, would have impact on the enzyme activity to a much lower extent than these external stimuli, providing broader applications in biocatalysts. On the other hand, such DNA-fueled DNA origami devices faced the drawbacks of low-enzyme loading yields (Grossi et al., 2017) and less controllable spatial arrangements of enzymes (Xin et al., 2013), limiting their application for a wide range of enzymes (Rajendran et al., 2017). Therefore, there is still a demand on the design of reconfigurable DNA nanocarrier that enables the efficient shape transformation and the high enzyme encapsulation yield without showing a harmful effect on the enzyme activity.

In this study, a 3D DNA scaffold (Douglas et al., 2012) was constructed to enable an efficient dynamic shape transition and applied for encapsulation of an enzyme xylitol dehydrogenase (XDH). Transformation of the open 2D-like scaffold with two conjunct domains to the closed 3D scaffold with two domains folded together was induced by short single-stranded DNA (linkers) hybridizing with both the edges of two domains. The closing process was monitored by the changes of fluorescence resonance energy transfer (FRET) with the variation in linker concentrations and hybridization temperatures. Typically, the DNA scaffold in the open state was transformed to its closed state in over 90% yield at a 1:1 molar ratio of DNA scaffold to linkers at 25°C for 12 h. This condition was applied for the encapsulation of enzyme. Xylitol dehydrogenase (XDH) was first assembled on the DNA scaffold in the open state with a high-enzyme loading yield, followed by the addition of the DNA closing keys to transform into the closed 3D scaffold. The enzyme encapsulated in the closed state exerted an activity comparable to that in the open state, ensuring that the catalytic activity of enzyme was maintained during the shape transformation process and upon encapsulation in the 3D DNA scaffold. The 3D DNA nanostructure with dynamic shape transformation would be applicable for the *in vitro* model of cellular dynamic process and the design of drug delivery, biosensing, biocatalytic, and diagnostic tools.

## Materials and Methods

### Materials

The single-stranded M13mp18 DNA scaffold (7249) was purchased from Guild Biosciences. pFN18A HaloTag® T7 Flexi® Vector and 5-chlorohexane (CH) derivative [HaloTag Succinimidyl Ester (O2) Ligand (P1691)] were purchased from Promega. Purified DNA origami staple strands, oligonucleotide primers, and all other oligonucleotides were obtained from Sigma-Aldrich (St. Louis, MO, United States), Japan Bio Services Co., LTD., (Saitama, Japan), or Thermo Fisher Scientific (Tokyo, Japan). *Escherichia coli* BL21(DE3)pLysS competent cells were purchased from Invitrogen (Carlsbad, CA, United States). *β*-Nicotinamide adenine dinucleotide in the oxidized form (NAD^+^) was obtained from Oriental Yeast (Tokyo, Japan). Xylitol, gel electrophoresis grade acrylamide, bis(acrylamide), and all other chemicals and reagents were purchased from Wako Chemicals (Tokyo, Japan) or Nacalai Tesque (Kyoto, Japan). Mini Elute Gel Extraction Kit was from QIAGEN (Tokyo, Japan). HisTrap HP column (5 ml), HiTrap SP XL column (5 ml), and Sephacryl S-400 were purchased from GE Healthcare Japan Inc., (Tokyo, Japan). PrimeSTAR HS DNA polymerase, T4 DNA ligase, and *E. coli* DH5α competent cells were obtained from TaKaRa Bio Inc., (Shiga, Japan). Ultrafree-MC-DV column was obtained from Merck Millipore (Darmstadt, Germany). Bio-spin® 6 column was purchased from Bio-Rad (Tokyo, Japan). Low-binding microtube (BT-150L, 1.5 ml, nonpyrogenic, and RNase-/DNase-free) was purchased from Ina OPTIKA CO. LTD., (Osaka, Japan).

### Expression of Enzyme HG-Xylitol Dehydrogenase

Enzyme HG-XDH (modular adaptor Halo-GCN4 fused xylitol dehydrogenase) was prepared as previously reported (Lin et al., 2021). Briefly, a gene encoding HG-XDH was constructed *via* overlapping PCR using p4LZ vector containing GCN4-XDH gene (Ngo et al., 2014) and pFN18A HaloTag® T7 Flexi® Vector containing a Halo-tag gene. The transformed *E. coli* BL21(DE3)pLysS competent cells were grown at 37°C until OD_550_ reached 0.45, and protein expression was induced with 1 mM IPTG for 24 h at 18°C. The soluble fraction of the cell lysate containing HG-XDH was loaded on a HisTrap HP column in 50 mM phosphate buffer (pH 7.5) containing 200 mM NaCl, 1 mM dithiothreitol (DTT), and 10 mM xylitol and was eluted by imidazole gradient. The fractions containing HG-XDH were loaded on a HiTrap SP XL column in 20 mM phosphate buffer (pH 7.0) containing 1 mM dithiothreitol and 10 mM xylitol and eluted by NaCl gradient. The purified HG-XDH was dialyzed by using 50 mM phosphate buffer (pH 8.0); containing 0.5 M NaCl, 1 mM dithiothreitol, 2 mM MgCl_2_, and 10 mM xylitol; and 50% glycerol, and stored at −20°C.

### Preparation of DNA Scaffold

DNA scaffold was prepared as previously described (Douglas et al., 2012; Amir et al., 2014). The solution (50 μL) contained M13mp18 (20 nM) and DNA staple strands (10 equiv, 200 nM; nucleotide sequences for DNA staple strands were shown in Supplementary Table S1) in a DNA scaffold folding buffer (pH 8.0) containing 5 mM Tris-HCl, 1 mM EDTA, and 8 mM MgCl_2_; the mixture was subjected to a thermal-annealing ramp for folding with following program: 80–60°C at 5 min/°C, 60–10°C at 75 min/°C, and finally holding at 10°C (C1000 Thermal Cycler, Bio-Rad). The sample was then purified by gel filtration (500 µL Sephacryl S-400) in an Ultrafree-MC-DV column with a buffer (pH 7.0) containing 40 mM Tris-HCl, 20 mM acetic acid, and 12.5 mM MgCl_2_ to remove the excess staple strands. The concentration of DNA scaffold was quantified by the absorbance at 260 nm (Nanodrop, Thermo Fisher Scientific Inc.,) using the determined extinction coefficient of DNA scaffold (1.20 × 10^8^ M^−1^cm^−1^) (Lin et al., 2021).

### Preparation of DNA Origami Scaffold Assembled With Enzymes

DNA scaffolds were constructed with three hairpin DNA-binding sites modified with 5-chlorohexane (CH) derivative for HG-XDH (Supplementary Figure S1 and Supplementary Table S2). 10 nM DNA scaffold with the binding sites was incubated with 200 nM HG-XDH in a buffer (pH 7.0) containing 40 mM Tris-HCl, 20 mM acetic acid, 12.5 mM MgCl_2_, 5 mM *β*-mercaptoethanol, 0.002% Tween20, and 1 µM ZnCl_2_ at 4°C for 1 h. The binding reaction mixture was purified by gel filtration (500 µL in volume of S-400) in an Ultrafree-MC-DV column with a buffer (pH 7.0) containing 40 mM Tris-HCl, 20 mM acetic acid, and 12.5 mM MgCl_2_ to remove the unbound proteins. The concentration of DNA scaffold–protein assembly was estimated from the absorbance at 260 nm by using the extinction coefficient of DNA scaffold (1.20 × 10^8^ M^−1^cm^−1^) (Lin et al., 2021).

### Closing Process of DNA Scaffold

The open state of DNA scaffold was first constructed with the six positions of linker strands left unhybridized, and then the corresponding six linker strands (Supplementary Table S3) were added in a 1:1 molar ratio. Typically, 5 nM DNA scaffold in the open state was hybridized with 5 nM DNA linker strands in the Microplate (Greiner Microplate, 96-well, PS, F-bottom (chimney well) µCLEAR®, black, nonbinding), with the buffer (pH 7.0) containing 40 mM Tris-HCl, 20 mM acetic acid, 12.5 mM MgCl_2_, and 0.002% Tween20 at 25°C for 12 h.

### Fluorescence Measurements and Fluorescence Resonance Energy Transfer Analyses

The nucleotide sequences of staple strands modified with Cy3 or Cy5 are shown in Supplementary Table S4. Fluorescence measurements were carried out on a microplate reader (TECAN Infinite® 200Pro). Fluorescence spectra of the samples were measured from 550 to 750 nm upon the excitation at 520 nm in the microplate (Greiner Microplate, 96-well, PS, F-bottom (chimney well) µCLEAR®, black, nonbinding) with five nm bandwidth. To study the kinetics of the closing process, the time courses of Cy3 fluorescence intensity (λ_em_ = 570 nm) and Cy5 fluorescence intensity (λ_em_ = 670 nm) excited at 520 nm as the optimal excitation wavelength of Cy3 were monitored.

### Calculation of Closing Efficiency (the Percentage of Closed Structures)

The closing efficiency of DNA scaffold was estimated by the Cy5 fluorescence intensity upon excitation of donor Cy3 at 520 nm in the fluorescence emission spectra after the closing process. The calculation followed the formula: *Y =* (*I*_HPO-control_−*I*_HPO + linkers_) / (*I*_HPO-control_−*I*_HPC-control_). Here, *I*_HPO-control_, *I*_HPC-control_, and *I*_HPO + linkers_ indicated the Cy5 fluorescence intensity of HPO-control, HPC-control, and HPO + linkers in the fluorescence emission spectra after 12 h incubation or hybridization, respectively. *Y* indicated the percentage of closed structures.

### Transmission Electron Microscopy Characterization

The DNA scaffold (2–3 nM, 2 μL) was placed onto a TEM grid and incubated for 2 min; then the extra sample was removed by a filter paper. A MilliQ water (15–20 μL) was used to wash the surface of TEM grid, followed by the incubation with 10% platinum blue (TI Blue) (4 µL) for 5 min. The surface was washed by the MilliQ water consecutively. Samples were analyzed by using a TEM microscope (JEOL JEM-2200FS + CETCOR).

### Atomic Force Microscopy Imaging and Statistical Analysis

The sample was deposited on a freshly cleaved mica (1.5 mm *ϕ*) surface and adsorbed for five min at ambient temperature, and then washed three times with a buffer (pH 7.0) containing 40 mM Tris-HCl, 20 mM acetic acid, and 12.5 mM MgCl_2_. The sample was scanned in the tapping mode using a fast-scanning AFM system (Nano Live Vision, RIBM Co., Ltd., Tsukuba, Japan) with a silicon nitride cantilever (Olympus BL-AC10DS-A2). At least three independent preparations of each sample were analyzed by AFM, and several images were acquired from different regions of the mica surface. The total number of DNA scaffolds corresponded to the well-formed structures observed under AFM. The binding of HG-XDH was counted for only HG-XDH bound to the perfectly folded DNA scaffold, and the quantification result is shown in Supplementary Table S5 (Lin et al., 2021).

### Enzyme Assay of HG–Xylitol Dehydrogenase

Catalytic activity of HG-XDH was measured by the changes of absorbance at 340 nm (25°C) deriving from the production of NADH on an Infinite 200 PRO microplate reader (TECAN). In a typical experiment, a reaction was started with an addition of NAD^+^ (2 mM) to a mixture of HG-XDH (2 nM dimer) and xylitol (300 mM) in a buffer (pH 7.0) containing 40 mM Tris-HCl, 20 mM acetic acid, 12.5 mM MgCl_2_, 100 mM NaCl, 1 μM ZnCl_2_, 5 µM BSA, and 0.002% Tween20. Enzyme activities were measured on the microplate (Greiner Microplate, 655901, 96-well, PS, F-bottom (chimney well) clear, nonbinding).

### Agarose Gel Electrophoresis

Conditions for the agarose gel electrophoresis were described in the figure captions. Typically, the samples were run on a 1% agarose gel in 1 × TAE (pH 8.0) containing 12.5 mM MgCl_2_ at 50 V for 6 h. The gel was visualized by using Molecular Imager FX pro (Bio-Rad) under ethidium bromide (EtBr) channel (λ_ex_ = 532 nm, λ_em_ = 605 nm), Cy3 channel (λ_ex_ = 532 nm, λ_em_ = 605 nm), or FRET channel (λ_ex_ = 532 nm, λ_em_ = 695 nm).

## Results and Discussion

### Construction and Characterization of DNA Scaffold

**FIGURE 1:**
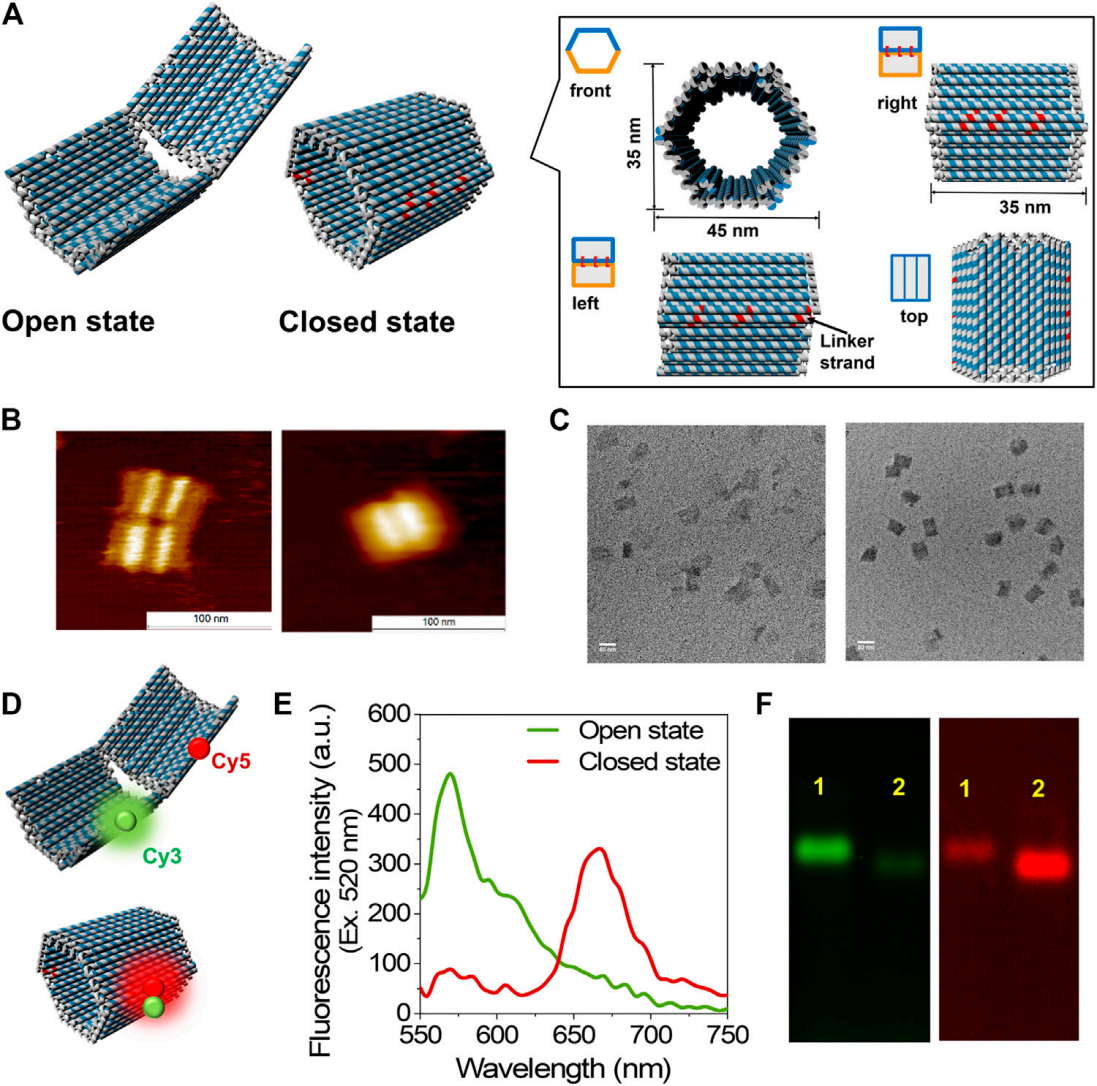
Construction and characterization of DNA scaffold. **(A)** The scheme of DNA scaffold in open and closed states; the dimensions and different views of closed state were shown in the box. The staple strands in red indicated the linker strands spanning and hybridizing with the two domains of DNA scaffold. **(B)** AFM characterization of DNA scaffolds, **left:** open state; **right:** closed state, scale bar: 100 nm. **(C)** TEM characterization of DNA scaffolds, **left:** open state; **right:** closed state, scale bar: 50 nm. **(D)** Models illustrate the Cy3 and Cy5 attachment positions on DNA scaffold. **(E)** Fluorescence emission spectra of open and closed states excited at 520 nm, conditions: 5 nM DNA scaffold was scanned in the buffer (pH 7.0) containing 40 mM Tris-HCl, 20 mM acetic acid, 12.5 mM MgCl_2_, and 0.002% Tween20. **(F)** Agarose gel electrophoretic analysis of DNA scaffolds. **Left:** Cy3 channel (λ_ex_ = 532 nm, λ_em_ = 605 nm); **right:** FRET channel (λ_ex_ = 532 nm, λ_em_ = 695 nm); **Lane 1:** Open state (HPO); **Lane 2:** Closed state (HPC). Agarose gel electrophoresis conditions: 1% agarose gel in 1 × TAE (pH 8.0) containing 12.5 mM MgCl_2_ at 50 V for 6 h.

The open and closed states of 3D DNA hexagonal prism (HP) (Douglas et al., 2012) were constructed in one step by DNA origami (Rothemund 2006), which consisted of two domains covalently attached in the rear by single-stranded scaffold hinges with a dimension of 35 nm × 35 nm × 45 nm in the closed state (Figure 1A and Supplementary Figure S1). Six types of single-stranded DNA linkers that hybridize with the complementary sequences spanning at both the edges of top and bottom domains of DNA scaffold were designed to fold the two domains together in the closed state (HPC). The six positions complementary to the linker sequences were left unhybridized for the open state (HPO) of DNA scaffold (Figure 1A). The resulting DNA scaffolds HPC and HPO were purified by size exclusion chromatography (Sephacryl S-400) to remove the excess staple strands and characterized by means of atomic force microscopy (AFM) and transmission electron microscopy (TEM) with estimated yields over 90% (Figures 1B,C, Supplementary Figure S2 and Supplementary Figure S3). The sizes of HPC were 35.4 ± 1.7 nm in length and 46.2 ± 1.3 nm in width, and those of HPO were 69.7 ± 4.8 nm in length and 43.5 ± 4.2 nm in width in the TEM images, which were consistent with the designed dimensions (Figure 1A and Supplementary Figure S4). Successful formation of the DNA scaffolds was also verified by agarose gel electrophoretic analyses. Over 90% of each DNA scaffold migrated as a unique band. HPC migrated faster than HPO possibly due to its compact closed structure (Supplementary Figure S5).

FRET (Roy et al., 2008) for each state was investigated to further identify the open and closed states of DNA scaffold. A pair of Cy3 (donor fluorophore) and Cy5 (acceptor fluorophore) was attached at the edge of each domain of DNA scaffold with the theoretical distance of 25 nm in the fully open state and within 1 nm in the closed state (Figure 1D). Fluorescence emission spectra of HPO (curve in green) and HPC (curve in red) upon the donor excitation (λ_ex_ = 520 nm) at 25°C are shown in Figure 1E. The primary emission of the donor (Cy3) alone at 570 nm in the open state indicated the far distance of two fluorophores, while the dominant peak at 670 nm in the closed state, corresponding the acceptor (Cy5) fluorescence emission, implied the efficient energy transfer between two dyes in the closed state. The difference in the efficiency of FRET for both the states was also supported by the agarose gel electrophoretic analysis. The band corresponding to the open state showed a stronger band intensity than the closed state in the Cy3 channel (λ_em_ = 605 nm); conversely, the band corresponding to the closed state exhibited a stronger band intensity than the open state in the FRET channel (λ_em_ = 695 nm) under the gel scanner (Figure 1F).

### Dynamic Shape Transformation of the DNA Scaffold

**FIGURE 2:**
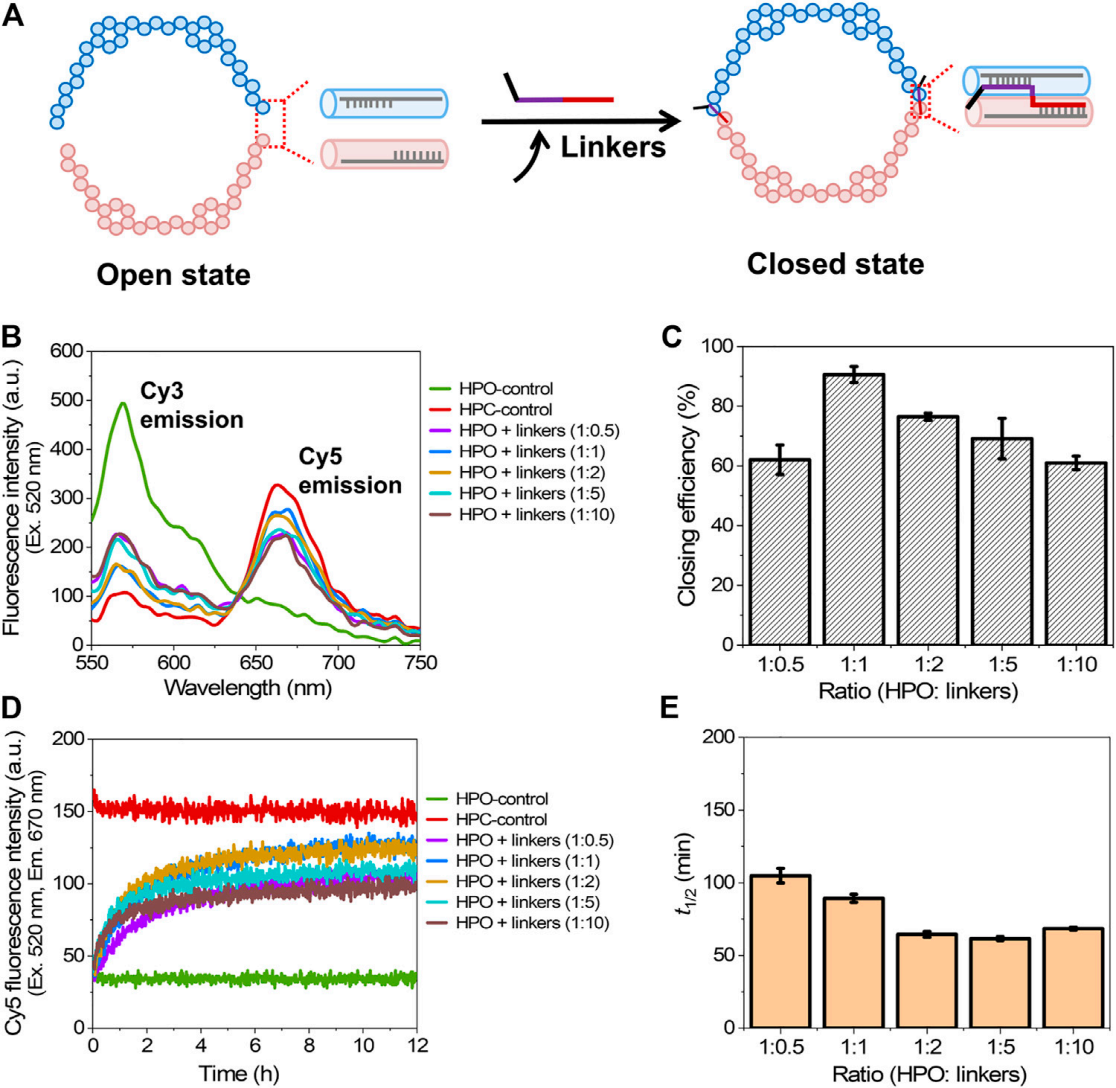
Closing process of the DNA scaffold. **(A)** A schematic representation of the closing mechanism through hybridization of the closing linkers to both the facing edges of two domains. Six types of single-stranded linkers were utilized to fully close the scaffold. **(B)** Fluorescence emission spectra (λ_ex_ = 520 nm) after incubation for 12 h with various molar ratios of the closing linkers at 25°C. **(C)** Effect of the molar ratio of HPO to the closing linkers (HPO: linkers) on the closing efficiency at 25°C. **(D)** Time course changes of the Cy5 fluorescence intensity (λ_ex_ = 520 nm, λ_em_ = 670 nm) during the closing process at various molar ratios of linkers at 25°C. **(E)** Half time (*t*_1/2_) of the closing process in **(D)**. Hybridization conditions: 5 nM HPO was incubated with 2.5–50 nM linkers in the buffer (pH 7.0) containing 40 mM Tris-HCl, 20 mM acetic acid, 12.5 mM MgCl_2_, and 0.002% Tween 20 at 25°C for 12 h.

The shape transformation of DNA scaffold from the open state (HPO) to the closed state (HPC) was initiated by the addition of six types of single-stranded short DNA strands (closing linkers) that hybridized to both the facing edges of two domains of the DNA scaffold (Figure 2A) (Douglas et al., 2012). The authentic open and closed states were prepared separately to apply for the control samples of HPO and HPC (HPO-control and HPC-control), respectively. Upon addition of the closing linkers, the Cy3 fluorescence intensity (λ_em_ = 570 nm) was decreased with the increase of Cy5 fluorescence intensity (λ_em_ = 670 nm), when excited at 520 nm (Figure 2B). The closing efficiency was estimated from the Cy5 fluorescence intensity after 12 h hybridization; the detail was shown in Materials and Methods. The molar ratio of HPO to the closing linkers was varied to investigate the efficiency of converting HPO to HPC. The optimal molar ratio (HPO: closing linkers) was found at 1:1, where the yield of the closed state reached 91% at 25°C for 12 h (Figure 2C). With the higher molar ratio, the closing yield was lowered because each of the hybridizing sites in two domains shared by a single closing linker was occupied by two molecules of the closing linker (Supplementary Note S1). In the molar ratio of 1:10, the closing yield was reduced to 61% (Figure 2C).

To study the kinetic aspect of closing process, a time course of Cy5 fluorescence intensity change was monitored (Figure 2D). The half time (*t*_1/2_) for shape transformation was estimated from the time-course changes. Interestingly, *t*_1/2_ gradually decreased with the molar ratio changed from 1:0.5 (105 min) to 1:2 (64 min), and kept in the similar value with 1:5, then slightly increased at 1:10 (68 min) (Figure 2E). These results suggested that the increase of the molar ratio to a certain range enhanced the hybridization kinetics of closing process, but at the same time, impeded the yield of closed state. The result was consistent with the previous report that reducing the DNA concentration decreased the rate of duplex formation in the DNA hybridization process (Markegard et al., 2016).

**FIGURE 3:**
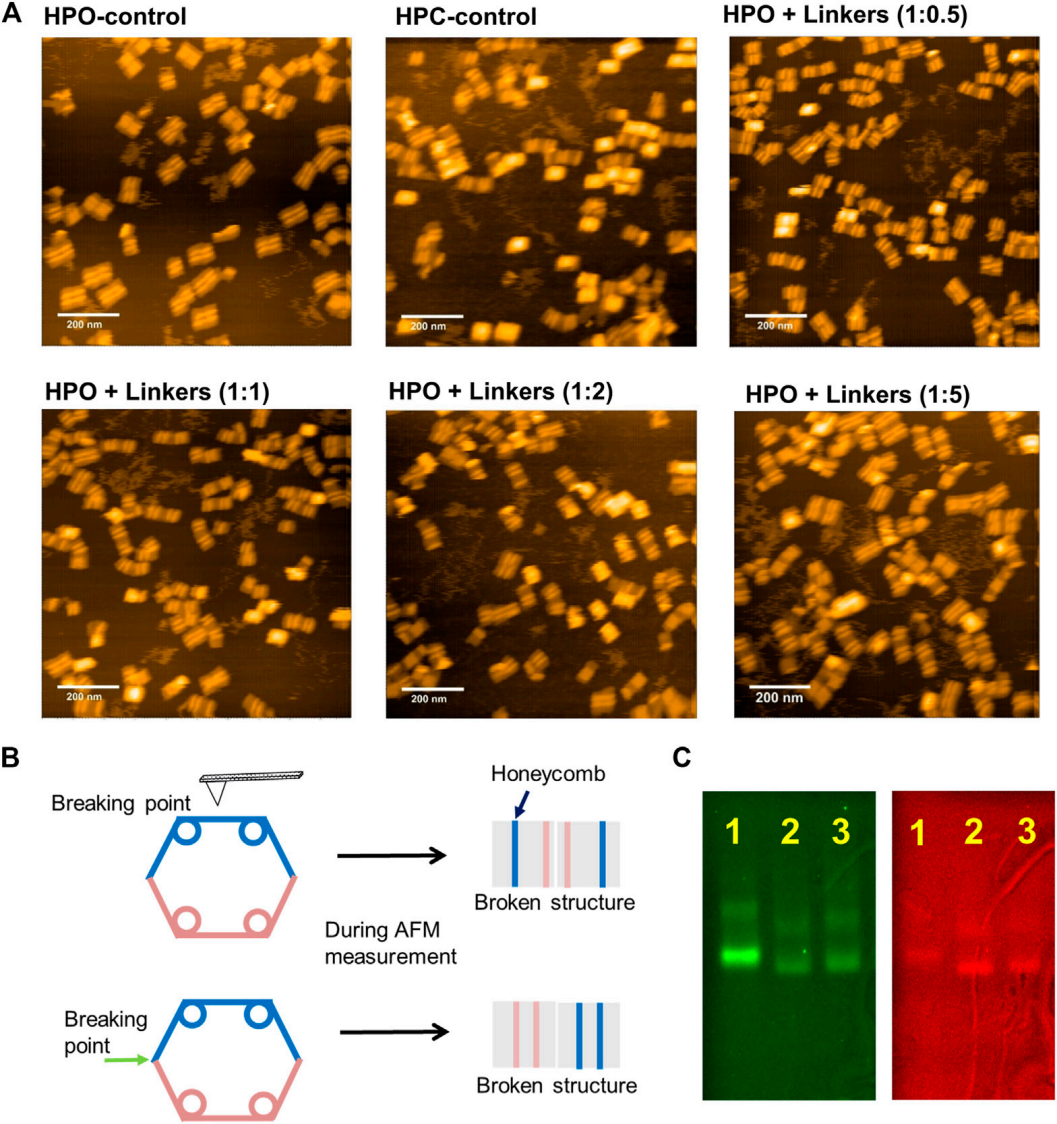
Characterization of the closing efficiency of DNA scaffold by AFM images and agarose gel electrophoresis. **(A)** AFM images of HPO-control, HPC-control, and HPO closed by linkers in the indicated molar ratio after 12 h incubation at 25°C. The closing yields of HPO + linkers (1:0.5), HPO + linkers (1:1), HPO + linkers (1:2), and HPO + linkers (1:5) were estimated to be 73% (124/169), 86% (174/203), 81% (116/143), and 75% (103/138) by AFM images, respectively. **(B)** A schematic representation of the closed structures systematically broken by the AFM cantilever during measurement (details were shown in Supplementary Figure S3 of supporting information). Images corresponding to the systematically broken structures were included to estimate the yield of closed structure. **(C)** Agarose gel electrophoretic analysis of the DNA scaffolds. Left: Cy3 channel (λ_ex_ = 532 nm, λ_em_ = 605 nm); right: FRET channel (λ_ex_ = 532 nm, λ_em_ = 695 nm); Lane 1: HPO-control; Lane 2: HPC-control; Lane 3: HPO + linkers (1:1, after 12 h hybridization at 25°C). Agarose gel electrophoretic analyses were conducted with 1.5% agarose gel in 1 × TAE (pH 8.0) containing 12.5 mM MgCl_2_ at 100 V for 6 h.

The closing yield estimated from the fluorescence intensity was supported by AFM analysis of these samples (Figure 3A). The closed structures were easily, but systematically, broken by AFM cantilever during the measurement as illustrated in Figure 3B and Supplementary Figure S3; thus, the systematically broken structures were counted as the closed structures. After 12 h incubation or hybridization at 25°C, the percentages of closed structures of HPC-control, HPO + linkers (1:0.5), HPO + linkers (1:1), HPO + linkers (1:2), and HPO + linkers (1:5) were estimated to be 96% (624 closed structures/total of 650 structures), 73% (124/169), 86% (174/203), 81% (116/143), and 75% (103/138) by AFM images, respectively. These yields were consistent with the results obtained from the FRET analyses. Closing yields were also verified by agarose gel electrophoretic analyses. The sample of HPO + linkers (1:1) showed a comparable mobility and band intensities in both the Cy3 and FRET channels with those of HPC-control, indicating the almost quantitative yield for the transformation to the closed state of DNA scaffold (Figure 3C). DNA origami comprises a high density of negatively charged phosphates on the DNA backbone. Upon addition of linkers, the closing process requires overcoming the electrostatic repulsion from the opposing domains of the DNA scaffold, which may explain the reason why the closing yield of HPO + linkers system was lower than 100%.

**FIGURE 4:**
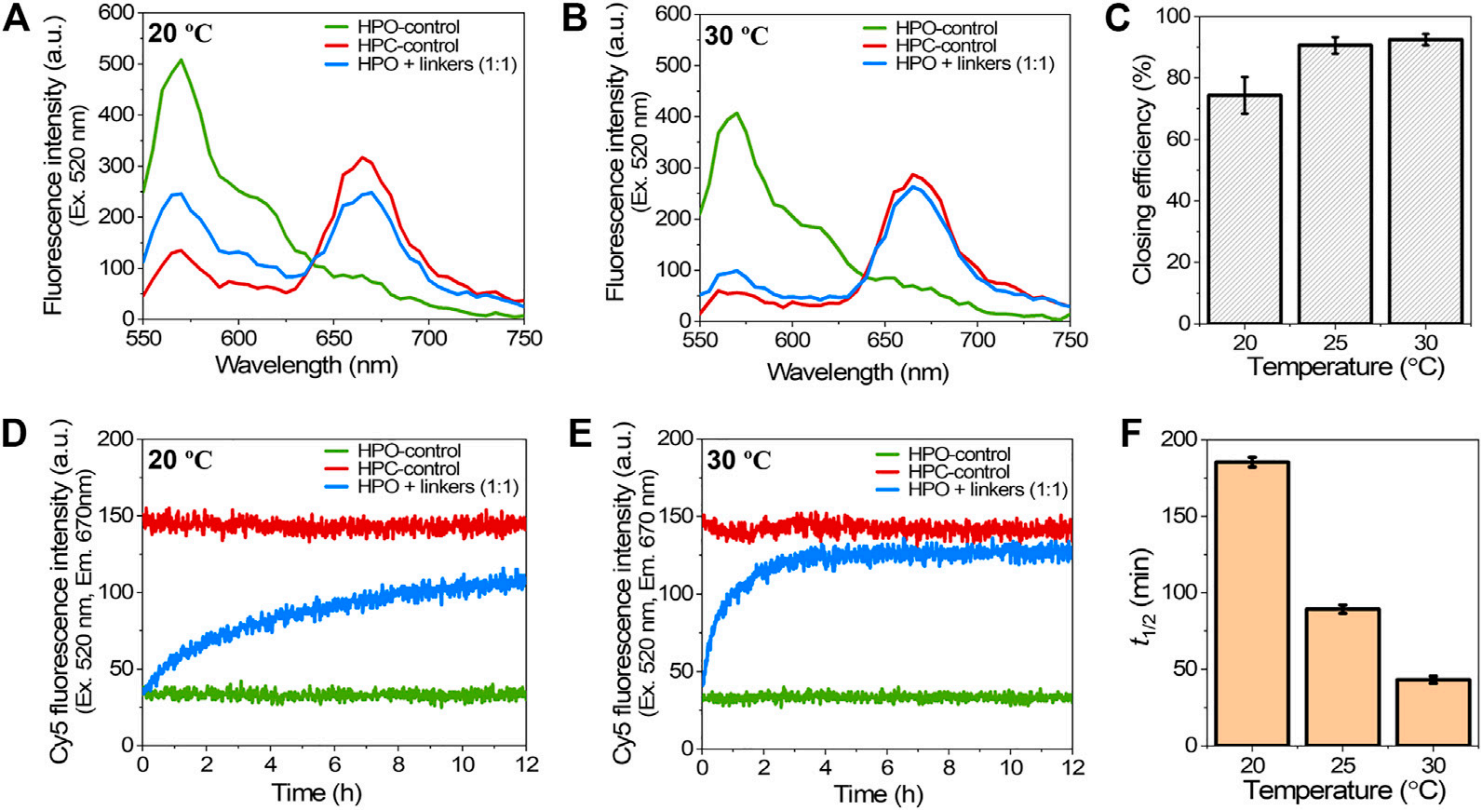
Effect of the hybridization temperature on the closing efficiency of DNA scaffold. **(A)** Fluorescence emission spectra (λ_ex_ = 520 nm) after incubation with 1:1 molar ratio of closing linkers for 12 h at 20°C. **(B)** Fluorescence emission spectra after incubation with 1:1 molar ratio of the closing linkers for 12 h at 30°C. **(C)** Effect of the hybridization temperature on closing efficiency at 1:1 molar ratio. **(D)** Time course changes of the Cy5 fluorescence intensity (λ_ex_ = 520 nm, λ_em_ = 670 nm) during the closing process with 1:1 molar ratio of the closing linkers at 20°C. **(E)** Time course changes of the Cy5 fluorescence intensity during the closing process with 1:1 molar ratio of the closing linkers at 30°C. **(F)** Summary of the half time (*t*_1/2_) of the closing process in **(D)**, **(E)**, and Figure 2D. Hybridization conditions: 5 nM HPO was incubated with 5 nM closing linkers in the buffer (pH 7.0) containing 40 mM Tris-HCl, 20 mM acetic acid, 12.5 mM MgCl_2_, and 0.002% Tween20 at the indicated temperature.

The effect of temperature on the hybridization of closing linkers during the closing process was investigated at the optimal molar ratio of HPO to closing linkers (1:1) by varying the incubation temperature from 20C to 30°C. The higher temperatures for hybridization resulted in the higher closing yields with accelerated closing kinetics. By comparing the fluorescence emission spectra after 12 h hybridization at 20°C and 30°C, the fluorescence emission spectrum of HPO + linkers at 30°C was much more approached to that of HPC-control (Figures 4A,B). The closing yields at 20, 25, and 30°C were 74, 91, and 93%, respectively, suggesting that the effect of temperature on closing yield was more profound for the temperature change from 20 to 25°C than that from 25 to 30°C (Figure 4C). Elevating the temperature significantly accelerated the hybridization kinetics (Figure 2D and Figures 4D,E) to shorten the half time (*t*_1/2_) from 185 min at 20°C to 89 min at 25°C, and to 43 min at 30°C (Figure 4F), which was consistent with the previous reports (Markegard et al., 2016; Groeer and Walther 2020). These results suggested that the molar ratio and the hybridization temperature played critical roles in the closing yield and the kinetics of closing process.

### Encapsulation of Enzyme into the DNA Scaffold and Evaluation of Enzyme Activity

**FIGURE 5:**
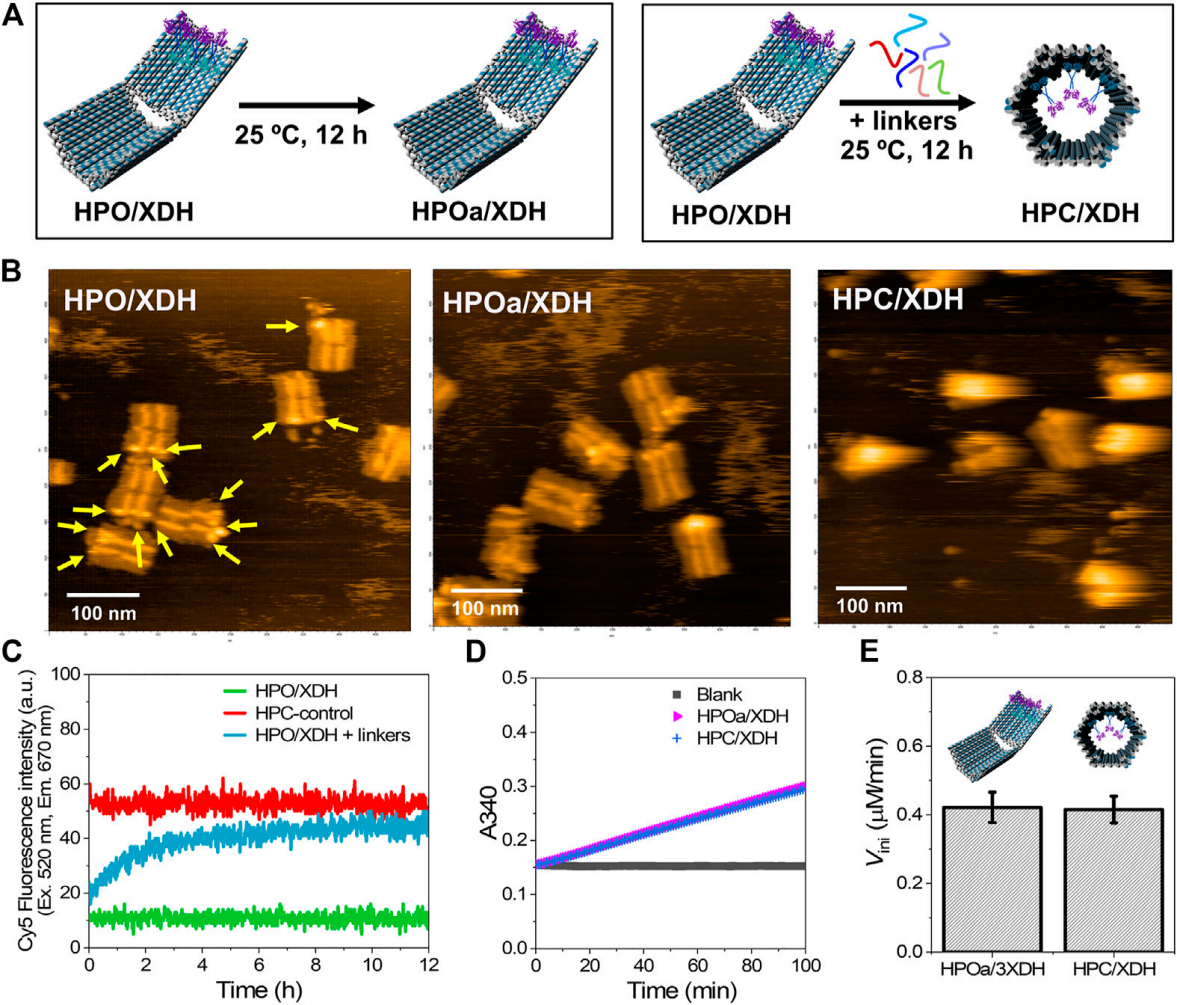
Xylitol dehydrogenase (XDH) encapsulated in HPC maintained its catalytic activity. **(A)** Schemes representing the sample incubation or hybridization with the closing keys of DNA scaffolded HG-XDH. HPO/XDH was incubated in the absence or presence of closing linkers at 25°C for 12 h to obtain a control sample for XDH assembled on the open state (HPOa/XDH) or encapsulated in the closed state (HPC/XDH). **(B)** Typical AFM images of HPO/XDH **(left),** HPOa/XDH **(middle),** and HPC/XDH **(right);** scale bar: 100 nm. The yellow arrows indicated the immobilized enzyme HG-XDH. **(C)** Time course changes of the Cy5 fluorescence intensity (λ_ex_ = 520 nm, λ_em_ = 670 nm) for the HPO/XDH closing process. Hybridization conditions: 1.5 nM HPO/XDH (scaffold concentration) was hybridized with 1.5 nM closing linkers in the buffer (pH 7.0) containing 40 mM Tris-HCl, 20 mM acetic acid, 12.5 mM MgCl_2_, 0.002% Tween20, and 5 µM BSA at 25°C for 12 h. **(D)** Time course changes of the absorbance at 340 nm (A340) for the enzyme reactions of HPOa/XDH and HPC/XDH. 2 nM HG-XDH (dimer) were reacted with 2 mM NAD^+^ and 300 mM xylitol in the buffer (pH 7.0) containing 40 mM Tris-HCl, 20 mM acetic acid, 12.5 mM MgCl_2_, 5 μM BSA, 1 µM ZnCl_2_, 100 mM NaCl, and 0.002% Tween20 at 25°C. **(E)** Comparison of the initial reaction velocities of HPOa/XDH and HPC/XDH **(D)**.

Xylitol dehydrogenase (XDH) (Watanabe et al., 2005), the second enzyme in the D-xylose metabolic pathway that converts xylitol to xylulose by consuming a cofactor NAD^+^, was assembled on the dynamic DNA scaffold (Figure 5A). The modular adaptor (Nakata et al., 2015; Ngo et al., 2016; Nguyen et al., 2017; Nguyen et al., 2019) stably locates an enzyme of interest at the specific position on DNA scaffold with a covalent linkage between the protein and the scaffold. XDH was fused to the C-terminal of modular adaptor (HG) consisting of the basic leucine zipper protein GCN4 (Ellenberger et al., 1992) and Halo-tag (England et al., 2015) to construct a fusion enzyme HG-XDH as reported previously (Lin et al., 2021). HG-XDH specifically reacts with the Halo-tag substrate 5-chlorohexane (CH) incorporated near the GCN4-binding DNA sequence (Nguyen et al., 2017; Nguyen et al., 2019). The dynamic DNA scaffold in the open state was constructed with three hairpin DNAs containing the GCN4-binding DNA sequence modified with CH for HG-XDH (Supplementary Table S2). The DNA scaffold with the HG-XDH binding sites was incubated with HG-XDH at 4°C for 1 h. The binding reaction mixture was purified by size exclusion chromatography (Sephacryl S-400) to remove the unbound HG-XDH and to obtain the purified DNA-enzyme assembly (HPO/XDH) (Lin et al., 2021). The details of experimental procedure were described in the Materials and Methods. The assembly yield of HG-XDH on DNA scaffold was estimated from the AFM images for each preparation of the samples (Figure 5B). In a typical preparation, 2.53 molecules of HG-XDH dimer were loaded on the three loading sites of each DNA scaffold in the open state (HPO) (Supplementary Table S5).

The resulting HPO/XDH was incubated in the presence or absence of closing linkers (1:1) to obtain the closed state encapsulating the enzymes (HPC/XDH) or HPOa/XDH, a control sample for XDH assembled on HPO treated with the same incubation time and temperature for the closing process (Figures 5A,B). The time course of the closing process for HPO/XDH monitored by the Cy5 fluorescence intensity indicated 92% closing yield after incubating for 12 h with a half-time value (*t*_1/2_) of 88 min (Figure 5C), which was similar to that of the HPO scaffold (89 min) at 25°C (Figure 2D). Formation of the closed structures was independently verified by AFM images with the estimated closing yield of 90% for HPO/XDH (Figure 5B). These results indicated that enzyme-loaded HPO scaffold was efficiently transformed to the enzyme-encapsulated HPC scaffold.

Enzyme reactions were investigated after the closing process. The reaction of HG-XDH on DNA scaffold was monitored spectrophotometrically by the production of NADH at 340 nm (Figure 5D). The comparable initial reaction velocities of HPOa/XDH and HPC/XDH indicated that HG-XDH encapsulated in the hexagonal prism nanocarrier HPC maintained a catalytic activity comparable to that assembled on the 2D-like DNA scaffold HPO (Figure 5E).

In our recent study (Lin et al., 2021), XDH or xylose reductase (XR) individually scaffolded on HPO fixed to the open state showed higher activity than the respective free enzyme. Indeed, the catalytic enhancements have been observed for a wide range of DNA–enzyme complexes with the proposed mechanisms of ordered hydration layer (Zhao et al., 2016), reduced adsorption (Timm and Niemeyer 2015), and substrate attraction (Lin and Wheeldon 2013). Besides, it has been proposed that the local pH change induced by the high, negative surface charge density of DNA nanostructures contributes the enhanced activity of enzyme scaffolded on the DNA nanostructure (Zhang et al., 2016). While we have directly observed a slight pH change near the surface of the DNA nanostructure, such local pH changes would not account for the higher activity of scaffolded enzymes because the optimal pH profiles of XDH and XR are pH 8.0 and pH 6.0, respectively. Likewise, contrary to the previous proposal, the neutral or net negative charge of substrates and cofactors for XDH and XR indicated that neither the HPO surface–substrate nor the HPO surface–cofactor electrostatic attractive interaction contributed for the enhancement of catalytic activity. We have also observed the preserved stability and prevention of adsorption on the reaction vessel for the HPO scaffolded enzyme, but these are not the determining factors for enhancing the activity of the scaffolded enzyme. Instead, it is likely that the ordered hydration layer formed by the high, negative surface charge density of the DNA nanostructure plays an important role. Zhao et al. have observed 4- to 10-fold higher turnover numbers of five DNA cage encapsulated enzymes (HRP, GOx, MDH, G6pDH, and LDH) than the free enzymes with the hypothesis that the hydration layer stabilized the enzyme (Zhao et al., 2016). In addition to the stabilization effect, we further propose that the hydration layer may enrich the local concentration of hydrophilic substrates; the relevant study is in progress at our laboratory. While the exact working mechanisms of catalytic enhancement of DNA scaffolded enzymes are still debating, it is believed scaffolds with high density of DNA helices create a favorable microenvironment for enzymes (Rudiuk et al., 2012). Such local microenvironment has been recently demonstrated by different biomolecular interactions on the DNA origami surface (Zhang et al., 2019; Huang et al., 2020).

In this work, the rigid 3D structure and large encapsulating capacity of the DNA scaffold in the closed state provide a sufficient space for XDH. The enzyme would contact the assembled surface, but unlike the nanocage system (Zhao et al., 2016), the enzyme resided far away from the other surface of the DNA scaffold. Such an environment of HPC could account for the comparable activities of XDH assembled in the open state and that encapsulated in the closed state. The characteristics of HPO and HPC are quite useful for the applications of the dynamic DNA scaffold as the nanocarrier for enzymes or other macromolecules to maintain the catalytic activity of assembled enzyme during the shape transformation, in which the catalytic activity of the enzyme on HPO is higher than its free form (Lin et al., 2021). Moreover, the enhancement of catalytic activity would be further tuned by assembling the same type of enzymes in the packed state (Dinh et al., 2020).

## Conclusion

In summary, this study presented the construction and characterization of a 3D DNA scaffold that undergoes a dynamic shape transition from the open state to the closed state induced by specific short DNA closing keys. Effects of the molar ratio for DNA scaffold to closing keys and the hybridization temperatures on the shape transformation were investigated. The optimal molar ratio of HPO to closing linkers was found at 1:1, where the closing state was obtained in over 90% yield at 25°C. Hybridization at the higher temperature resulted in the higher closing yield with an acceleration of closing kinetics. The efficient shape transformation of DNA scaffold was applied for an enzyme encapsulation with high loading yield. The activity of efficiently encapsulated xylitol dehydrogenase in the closed state was comparable to that in the open state after the same closing process. The fact that the individual enzyme activity was maintained upon encapsulation in the hexagonal prism nanocarrier HPC supports further applications of the present system not only for the enzyme nanocarrier but also for the drug delivery, biosensing, and diagnostic tools.

## Data Availability

The original contributions presented in the study are included in the article/Supplementary Material, and further inquiries can be directed to the corresponding author.
