# Supplementary material for: Dynamic Shape Transformation of a DNA Scaffold Applied for an Enzyme Nanocarrier
Source: Front Chem. 2021 Jun 24;9:697857. doi: 10.3389/fchem.2021.697857 (PMC8263910; doi:10.3389/fchem.2021.697857)
Supplement: Supplementary file 1 [file DataSheet1.PDF]

Supplementary Material

Dynamic shape transformation of a DNA scaffold applied for an enzyme nanocarrier

Peng Lin[1], Huyen Dinh[1], Eiji Nakata[1], Takashi Morii*, [1]

[1]Institute of Advanced Energy, Kyoto University, Uji, Kyoto 611-0011, Japan

***Correspondence:**
Takashi Morii
t-morii@iae.kyoto-u.ac.jp

Table of contents

Note S1. The effect of molar ratio (HPO: linkers) on the closing efficiency …………………………... S3

Figure S1. Front view and blueprint modified from caDNAno software interface of DNA scaffold with DNA linker strands ………………………………………………………………………………………… S4

Figure S2. AFM images of DNA scaffold in open and closed states ………………………………………… S5

Figure S3. AFM images of closed structures broken by cantilever when measured by AFM ………………. S6

Figure S4. Typical TEM images of DNA scaffold in open and closed states ………………..……………… S7

Figure S5. Agarose gel electrophoresis of DNA scaffold in open and closed states …………………………. S8

Table S1. Unmodified staple strands used for the assembly of the DNA origami scaffold ……….. S9

Table S2. Nucleotide sequences for the staple strands containing the binding sites for HG-XDH with CH modification …………………………………………………………………………………......... S13

Table S3. Nucleotide sequences of linkers ……………………………………………………………..… S14

Table S4. Nucleotide sequences of staple strands modified with Cy3 or Cy5 ……………….…………… S14

Table S5. Average number of assembled enzyme on DNA scaffold……………………………………… S14

Supplementary references ………………………………………………………………………… S15

Note S1. The effect of molar ratio (HPO: linkers) on the closing efficiency

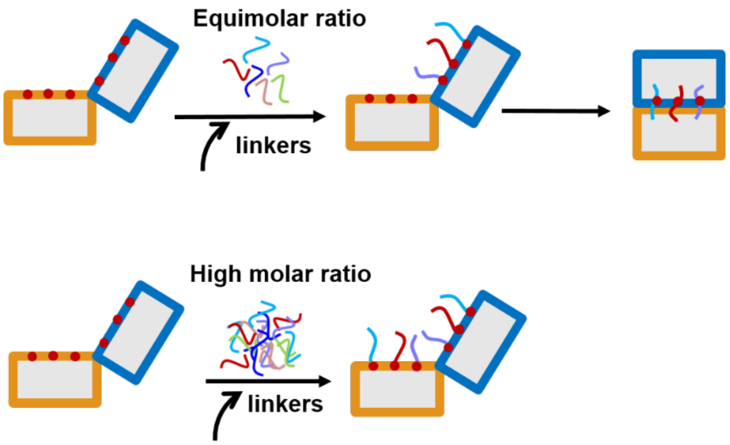

In equimolar ratio (HPO: linkers), DNA linkers were able to hybridize with of both domains and reached high closing efficiency, by contrast, in high molar ratio, the binding sites of both domains were saturated with the linkers, the low closing yield was obtained. The red circles on DNA scaffold indicated the positions to hybridize with the linkers.

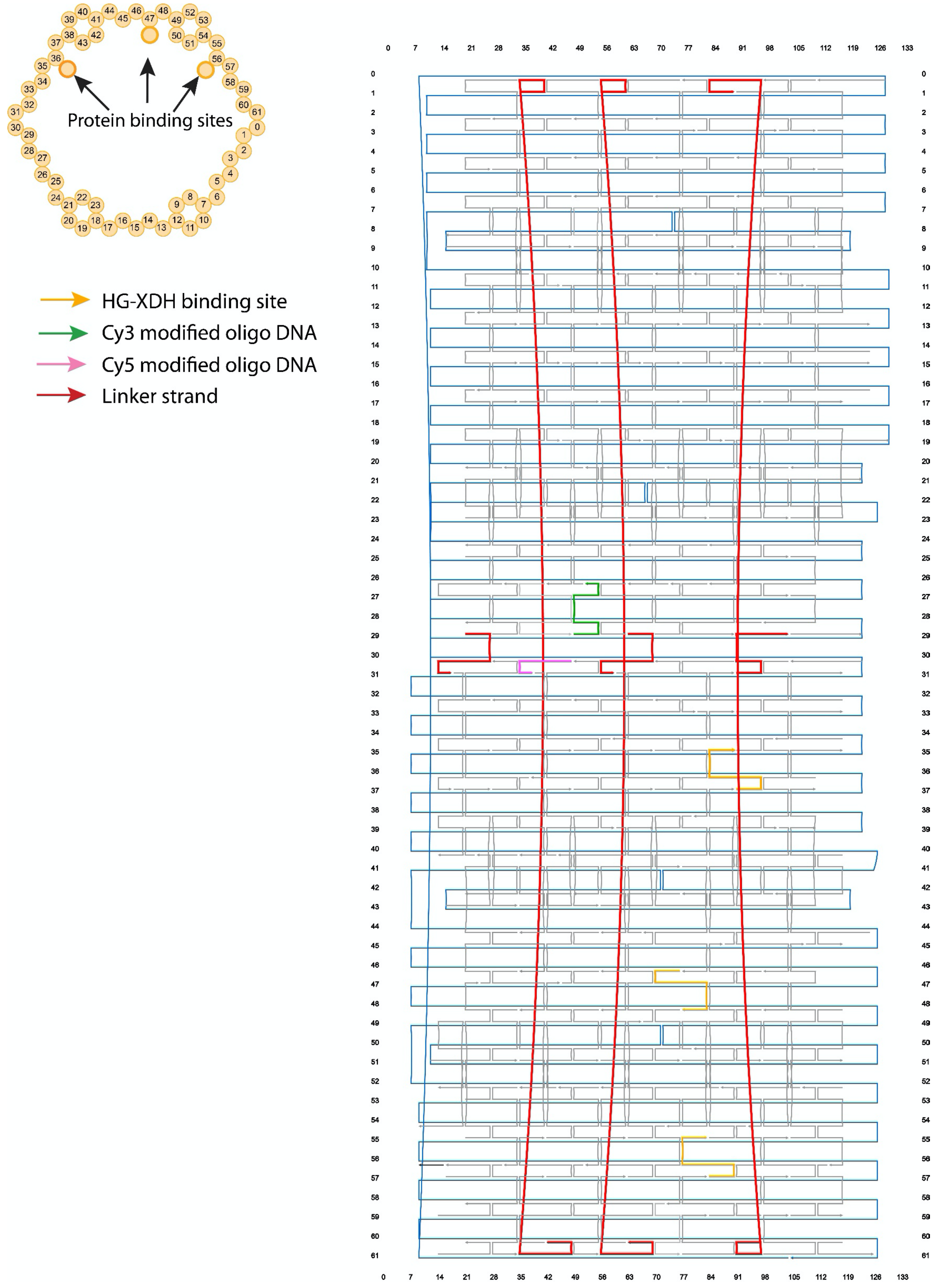

Figure S1. Front view and blueprint modified from caDNAno[S1] software interface of DNA scaffold (http://cadnano.org/) with linker strands. The staple strands shown in orange were enzyme HG-XDH binding sites. The staple strand shown in green shown in green was Cy3-modified oligo, the staple strand shown in pink was Cy5-modified oligo. The staple strands shown in red were linker strands of DNA scaffold.

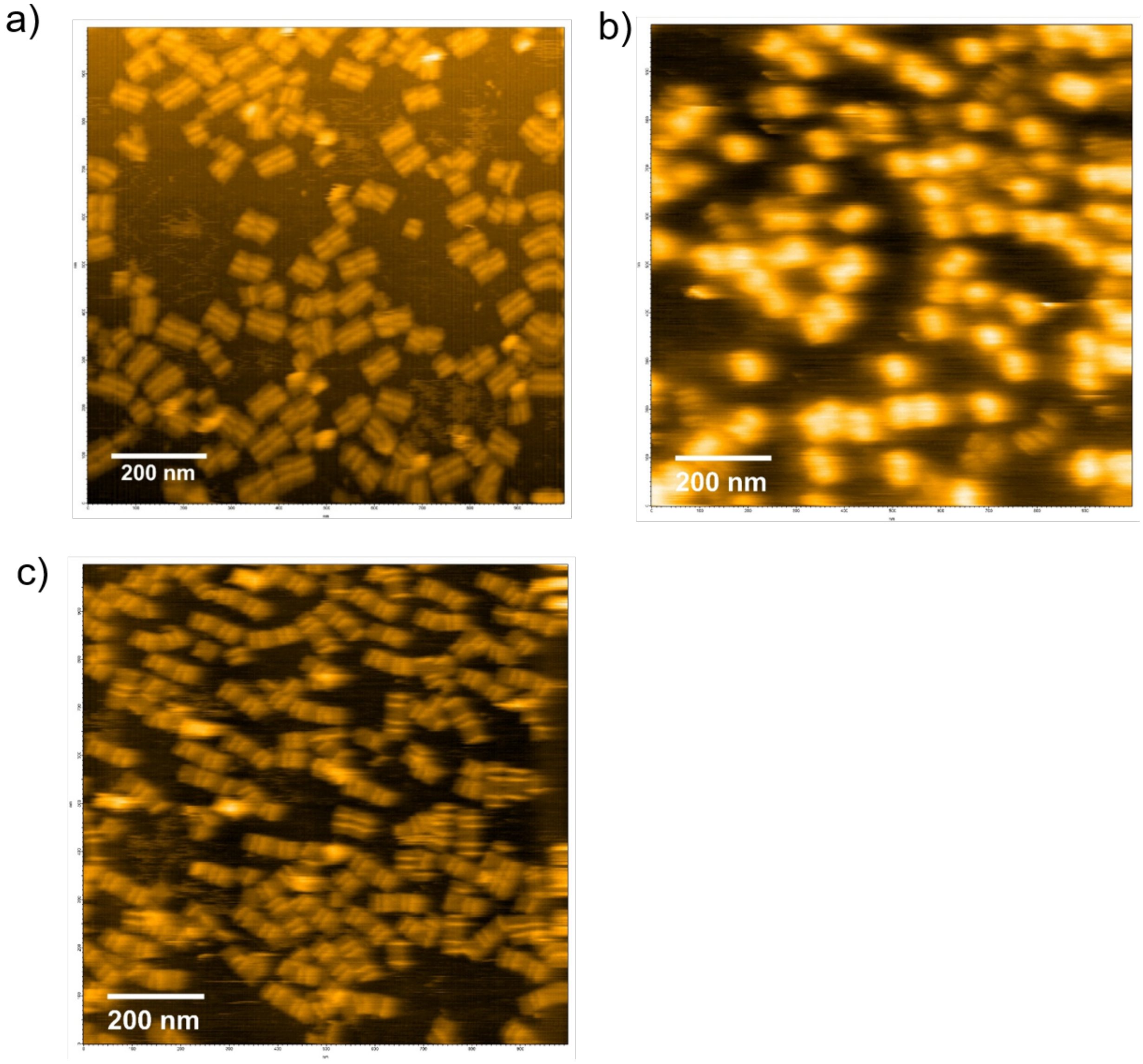

Figure S2. AFM images of DNA scaffolds in open and closed states. (a) AFM image of DNA scaffold in open state (HPO) with estimated 92% well-formed yield (276/300). (b) DNA scaffold in closed state (HPC) under soft measurement tapping. (c) DNA scaffold in closed state (HPC) under strong measurement tapping, the closed structures were broken by cantilever (the detail was shown in Figure S3). The well-formed yield of closed state was estimated to be 96% (624/650), the intact closed structures and broken structures were counted as the well-formed closed structures.

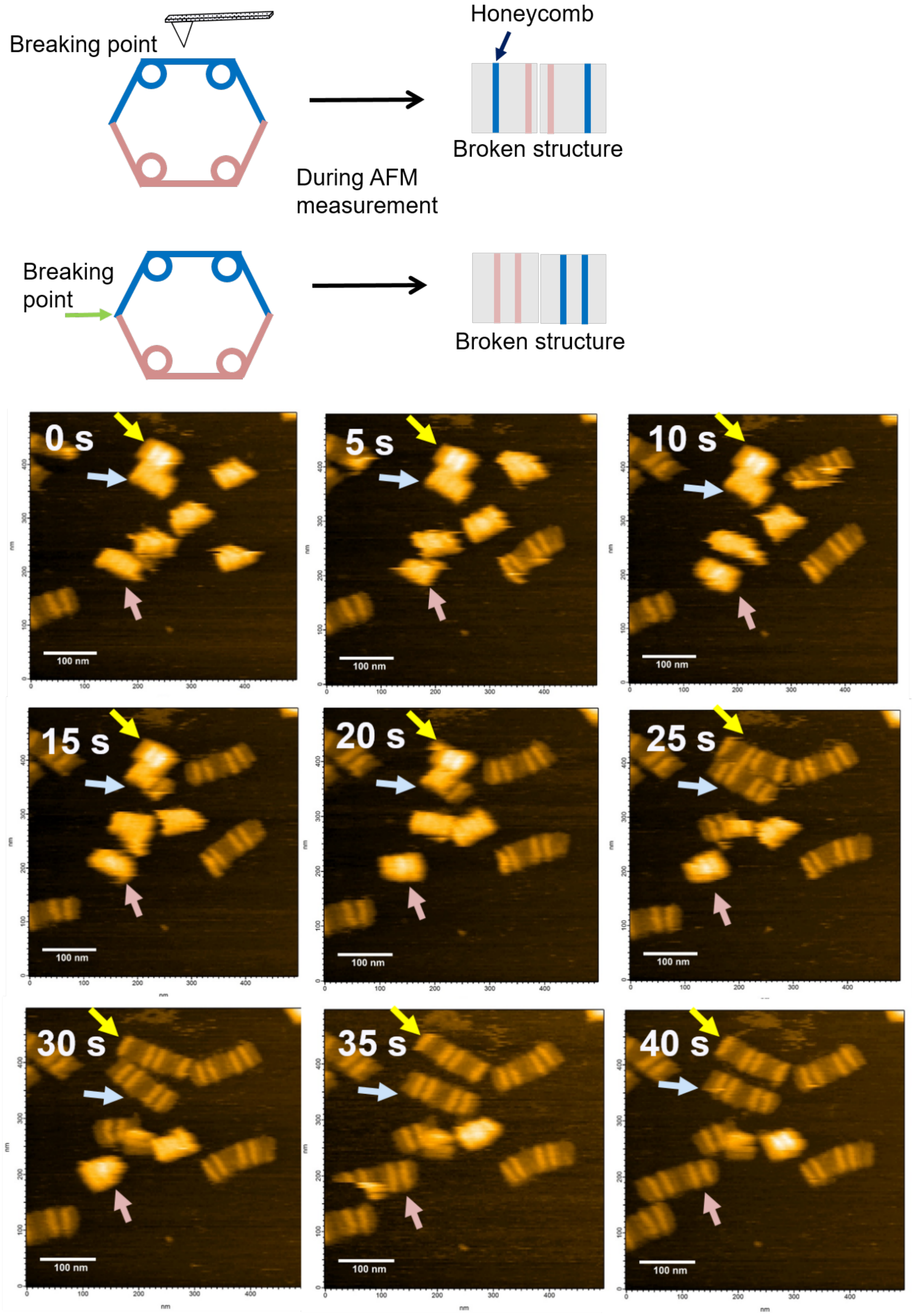

Figure S3. AFM images of closed structures broken by cantilever when measured by AFM. The arrows showed typical examples.

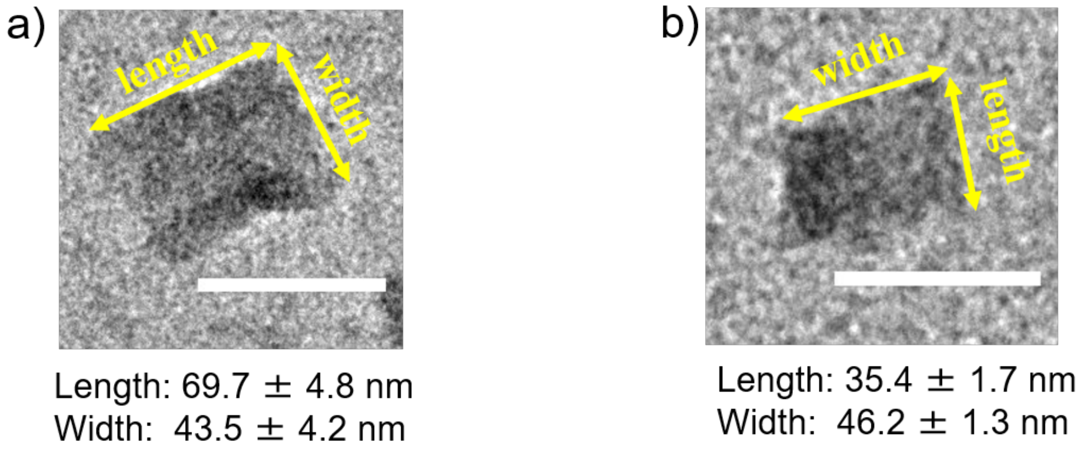

Figure S4. Typical TEM images of DNA scaffold in open and closed states. (a) TEM image of DNA scaffold in open state, scale bare: 50 nm. (b) TEM image of DNA scaffold in closed state, scale bar: 50 nm. The measured sizes of open state were 69.7 ± 4.8 nm in length and 43.5 ± 4.2 nm in width. The measured sizes of closed state was 35.4 ± 1.7 nm in length and 46.2 ± 1.3 nm in width. The well-formed yield of closed state was estimated to be 92% (187/203) by TEM images.

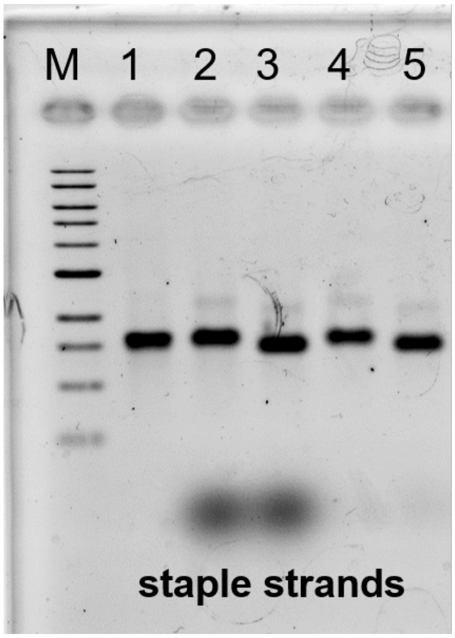

Figure S5. Agarose gel electrophoresis of DNA scaffold in open and closed states. Lane M: 1 kb DNA marker, Lane 1: single strand DNA template (M13mp18). Lane 2: DNA scaffold in open state before purification. Lane 3: DNA scaffold in closed state before purification. Lane 4: DNA scaffold in open state after S-400 purification. Lane 5: DNA scaffold in closed state after S-400 purification. Gel running conditions: 1% agarose, 1 × TAE buffer (pH 8.0) with 12.5 mM $MgCl_2$, 50 V, 6 h.

Table S1. Unmodified staple strands used for the assembly of the DNA origami scaffold

Oligo DNA	Sequence (from 5' to 3')
1	AAAATCCCTTATAATGCCAGCTGCATTACTCACTG
2	GATGGTGGTTCCGAGGCCAACGCGCGGGACTCACA
3	CAGCAGGCGAAAATGGTTTGCGTATTGGGGGTGCC
4	GAGAGTTTGAGACGGGCAACATCCACACAACATACGCTGTTT
5	TTTCTTTAAGCATAAAGTGTAGAATTCGTAATCATCTCTTCG
6	CCCGCTTACGGCCAGTGCCAAGCCAGGGTTTTCCCTGAGGGG
7	TTAATTGTGCCTGCAGGTCGAAAGGCGA
8	TAATGAGGGATCCCCGGGTACGGCGAAA
9	CTCAAGCCTGGCGCCAGTTTGCCC
10	ATAGAGCCGGTCACCAGGCAGCAA
11	CCTGTGTGGAAGGGCGATCGGAAAGCGC
12	TTAAGTTGGCCTCAGGAAGATTGGGATA
13	GGGGGATCAGCCAGCTTTCCGCAAACGG
14	CTATTACTTCTGGTGCCGGAACCGTCGG
15	ACGACGAAGATGGGCGCATCGGTTCTAGGATGAACGGTAA
16	CATTCGCTAAATGTGAGCGAGGCGTCTGATTCGCATTGTAAA
17	GGTCACGCCGGAGATCTGGAGGTACCCC
18	CGGATTGGAGAGATCTACAAAGTAATGT
19	ATTCTCCGAACGCCAATCAGCTTGTATA
20	GAGAATCCTGATAAGACAGTCAAATCACGGCAAAGAACA
21	CTGAGAGGGGTAGCAAAAGGGTGAGAAAAGCA
22	TATCAGGAAAGCCCCAAAAACGGGCGCGAATCGGTTGTACCATGCC
23	TGTTAAAGCCTTCCAAAATTTTTAGAACACTT
24	TCGTAAAACTAGCATGTCATAGCATTAATTAGCAAAATTAGGCC
25	GGTTGATCATCAATAAGCCTCAGAGCATAAG
26	AGCAAATGTTTAGCTATGACCCTGTAATCCT
27	CGTTAATTTCGCAACGGGAGA
28	GGAATTAATGTTGGTGTCAGTATCGGGTAACGCTTGCACGTTGCG
29	ATTCTATTTTTACCGTAACGCACTCGTGCTGCCTCTAGATGAGCTA
30	GTAGGTAAAAGCTAAGCTGAAGTGTCTGCCTTTAACAGA
31	TGACCAATAGGTGGGAAGCACCGCGCCAGCTCGAG
32	AATGCAAAAAACATTATATTTATAACAACAGGTCCAAA
33	CATAATAATTCTAACAACACCAGGCTGCGGGCGGTC
34	ATATCTACTATAAATATAAGAGGTCTTT
35	TTGATGGTCACTGCGAACCAGACCCGAA
36	TCCATGCTGTGCTTAGAACCATAATTAA
37	CATGTTTATAGTAGATCATATCAAACAA
38	AAGTACGAAGGTGGAATCAGATCATTGC
39	CATTCCATCATTTGAGGAAGATCATTTTTTAAGGC
40	CCCAATTATAACCTATTTAAATTAAATTTTTGTTAATCAAAATATTTTA
41	AGCTTCAAAGCGAACGAGTAGATTTAGT
42	ATCAGGTCATTTTTGCGGATGAGCTCAA
43	ACCATTGAATAAGGAATGGTAGAAGACG

44	TTATAGTTTGCTCCTTTTGATGCAACTA
45	AGCGCGGAATAGAGCAATAACGGATAGT
46	ATTGCATAGGATTAGAGAGTAGAAGTTT
47	AAGGACTGGATCGTTTAATCTACGGTTT
48	GGAAGCCGGAAGCAAACTCCAGTTGATT
49	AGAGGGGTAAAAACCAAGTCAGGATTGTGAAAGATGAA
50	ACAAATGCAGAGATTTATCAGTGAAAATCAA
51	AAGTTTTGCCAGAGCTTCAAATATCGCG
52	ACGCCAACCCCCTCAAATGCTATCAAAA
53	CATAGTACGTCATAAATATTCCTGACTA
54	ATAACCCTAGCGTCCAATACTAAAGCGG
55	ACGATAATAGTAAAATGTTTAATTAAGA
56	AGAAGAACCGGATATTCAGCGAAAACTTTTT
57	AAATCATCAAGAGTAATGAGGGTACAGAGGC
58	AATGGCGCATAGGCTGGGAACGAGCGACCTG
59	CCCATAAGGCTTGCCCTAGATTCAGAAGGCA
60	ACAAACACCAGAACGAGACAACATAAGAGGC
61	CCTTTGGGCTTGAGATGTTAATAAAAACACT
62	CCATTCAACTTTAATCACGTTGGGAGCGATT
63	CGGTGTATCATAAGGATAAAT
64	TTGAAAGAGGACTTACCTTATGCGATTTTAA
65	CATGAGGCCACTACTCAGTTGATACATA
66	TTTGAGGAAAACGATATTACATACGAGG
67	CTCCATGTACACTAAACGAACCACTATC
68	TGTGTCGGACCCCCAAGAAAACCAGACG
69	TTGTATCGCGCGAATATACCAAATAGCG
70	CGTAATGAAGTTTCTCGTCACGGAGTTA
71	CCAACCTACTAAAGGACAGCATTCGGTC
72	AAAAGAATTGGCTAGCAACACCGCCCAC
73	CATCTTTAAATCCGGCGCAGAGCCGACA
74	ATACCAAATCGCCTGGAACCGCAGCTTGATACCGATCGGTTT
75	GCTGAGGATAATAATTTTTTCAACAACT
76	GCATAACAAATCTCCAAAAAATTTCTGT
77	ATGACAACAAAAGGAGCCTTTTCCAGAC
78	GAATTTCTTAAAAACTGACCAACT
79	TAAAGGACGGAGTGAGAATAGACCCTCATTTTCAGGCCACCC
80	ATCAGCTGATCTAAAGTTTTGTGTAGCA
81	CAGATTGCGACTTGCAGCCTCAGCATTA
82	TTCAACAAAGCCCAATAGGAAAGAACCGCCACCCTTGAAACATGAAAGT
83	CTAACGTTGACGATATATCGGAACCTTG
84	ATGGGATACCGTAACACTGAGAGGTTTAGTACCGCGGCTGAG
85	GAATAAGGCTCCAACCATTTAGCCGCTGA
86	GTTAGTAACCAGTACAAACTAAGGTGTATCACCGTGGATTAGGATTAGC
87	TTCCACAGATATAAGTATAGC
88	CCGGAATCAACGCCTCGTCTTAATTGTATAGTTGCCGGTCAACAGA
89	ATTATTCCAGAACCGGATAGCGTTT

90	ATTAAGACACCCTCCCCATGTTTTG
91	AAGAGAAACTCAGGTTTCGTCAAT
92	ACAGGAGTGTACTGAATCCTCCTCCCTCCGGC
93	TGGCTTTGGAAAGCCAGAGCCCCTTATTCGTC
94	AGCCAGAACCCACCGTACAATGAATTATTTCAAAGACGCAA
95	CAGCACCACCAATCAAGTAGCAAGAAAATTCTGGC
96	ATACAGAACCCAGACTGCACC
97	CCGCCACATTGACAGGAGGTTCGTATAA
98	TCAGAGCGTCAGACGATTGGCAGTGCCT
99	CCACCCTTTCACAAACAAATAGTAATAA
100	AAAATCACCGGAACGCAGTCTCTGAATT
101	ATTGGGAATTATTCAACTACGCAGTAGCCGAACAC
102	ATAGCCCACCACCGGAACCGCATTAAAG
103	AGTCGCCAAATAGAAAATTTTAAGGTAATTTCCA
104	ACCAAGGTAAGGCATGAAGGAAACTTAGACGATAG
105	AGGTGAATTATCACAGCGTTTGCCATCT
106	TCAATAGGCCGGAAACGTCACATCAGTAGCGACAGCTCAGAG
107	TTACCAGAGCACCATTACCATTTTGCCTTTAGCGTGCCACCC
108	GGGCGACAGAGCCAGCAAAATTAGCGCGTTTTCATAGAGCCG
109	GGGAGGGGACTTGAGCCATTTTTCGGTC
110	AACATAGCTAGAGAGATCAGCCATCCTGAAT
111	AAGAACTATATTGACGGAAAT
112	GAAGAGTTAAATTTGCCCGAGCGT
113	AATAGCAATATAAAAGAAACGGTCACAA
114	GAAGCCCTACATACATAAAGGATATGGT
115	AAGCAGATATGTTAGCAAACGGACAAAA
116	TACCAGATTAAGACTCCTTATCGATTGA
117	CCTTGTTTAAAATCAAGATTAGTTTTCAATA
118	AATTGCACCCAGCTACAACGACGACGCCTGT
119	GATTTTTGAACAAACTTTCCTTATCATTCATTACC
120	AATGAAAGGAGAATCGGGTATTAAACCATTTT
121	CAGGCGGGAGGTTTTGATATAGAATTCATCG
122	TAAAGTTACAAAATAAAAACCCACCCTAATT
123	CTTACCAAGGTAAATAAGTCCTGAACAA
124	TATATTATTTATCCCAAGAGCGCTAACCAAT
125	GAACCTCTCTAAGAAACAAGCAAGCCGTAGTACCGAAGCGCACGAGGAA
126	GCAAGCAGGGCTTAATTGAGAAAATAAGAATAAACAACTATA
127	TTATCAAATGTAGAAATATCATCTTACC
128	GCGCCCAGCTAATGCGGCTGTGTCAGAGGAAAAGT
129	TAGGAATCCAAGAATAACTGAACAAAGT
130	ATTGGCTTATTAAAGCCAACGCTCTCATAATTACTAGACCGGCTT
131	ATAATATCCCATAAGAATTACAATGA
132	TACGAGCCAATAGAGTAATTCAATTTAG
133	CAATAATCAGAACGCAATAAATATTTAA
134	GCAGAGGTTTAATGGTTTGAAGAACGCG
135	CAACGCCCCGTGTGATAAATACAAATCC
136	GCCACAACATGGCTATTTAAGAAAC

137	TTATACAAATTCTTTGTTTAG
138	GAAAACAGTAAATCAGAAGCCTTACGTCAAA
139	GCCACCAGTACCGGTATCCGACTTCCTTTAC
140	AGAAAACCGCTATTAATTAATGGAAACA
141	AATCGCATAGAATCCTTGAAAATTTGAA
142	TGTAAATGATAGCTTAGATTATTAATTA
143	AGGTTGGGAGAAGAGTCAATAACAAACA
144	CGTTTTTTCAACCTAAACATTTTCCGACAAAACGC
145	CCTAGACAAAATACCGAAACATGTTGTCCAGATTT
146	GTACATATTGAGTATGGCAATCTTCTGA
147	TTACCTTCGGAACAATATTCCAGAACCT
148	CATTTAAAGTTGCTCCTGAGAATTTGCA
149	TCAAGAAACAAAATGGAGAAAAAAGAAA
150	AAGAAGATTATTCAGTAACAGAGGTTTA
151	AGACAATTTCACATAGCGCTGATGAGGCGTTATC
152	TCGTTGAATAATAGATAATACATTATCAATACCAG
153	TACATCGCGCGCAGACTAATAGATTAGAAAATCAATTAA
154	GATTGTTTGGCCACGCTGAGA
155	ATAATGGATTAACACATCACCTTGCTGACTTT
156	ACCATATAACAGAGATATCAAACCCTCATGAGGATCACC
157	CGTAAAACGAACCATCTGGTCAGTTGGCGCCG
158	TTGCGTAATCGCCACAGTTGAAAGGAATACTA
159	CGACAACTCTAAAGCCGCCTGAAAAGGGTGGA
160	ACACATTTTGTTTTAATTTTC
161	TATTAGAACCTCAAGTGAGGCAGAGATATTGA
162	TCACCAAGTTAACAAAAAGACGCTGTTATATACCG
163	ACAAGGCGAATGATGAAGTGAATTTTAACCTAAAA
164	TAGGAGCTGAGGAAGATAGCCGCTATTAATCC
165	TGAAAAATCGTATTTAAAAGTAATCAATAAAT
166	CAGTGAAAGCGCTCATGTTCTTTGACAG
167	AAACACAGACAGCCATTCACTTGCTAGAATCTTAATGC
168	CAGTAATCAACAGTGATTATATCATCAATATAATCTTAATTTAAATCCT
169	GGCCAACGGTCAGTAAGGGTTTGATTATCAGATGAACATTATAACAATT
170	TCTGACCAAGATAACAAAATTGCGGAATTATCATCAAGAAACTTAGAAG
171	TACGTGGTACCGAACAGAAATCAATAACGGAT
172	TTGAATGCTAAAACGATTTTCTACCTTT
173	TTAAAAGAGTCAGAATCCGGGCGCAGCCGGCGTTGTTC
174	CGCAATTAACTTTTAGAGTGTAGCGATTTAGAGTCCAC
175	AGATCAAACTCGTATAACGTACTA
176	GGCCACCGAGTATTTACATTGGCA
177	TCACGCATCAATCGTCTGAAAACATTCT
178	GCAATACGAAATACCTACATTGAACCCT
179	ATAACATGCAACAGGAAAAACGTAAGAA
180	GAAGAACACAATATTACCGCCAATATTT
181	GAGTAACCACGGAACCCGACTCCA
182	GAAAGCGAAAGGAGCTGAGAA

183	GCCGCGCAGAGCGGGAGCTAAATTAGTA
184	CAGGGCGCGTGCTTTCCTCGTCTGAGTA
185	CGGGGAATAGGGCGCTGGCAACAGGAACGGTACGCCTGTCCA
186	AGCCCCCGGTCACGCTGCGCGGCCGATTAAAGGGACGTTGTA
187	AGATAGGGTTGAGTGAACGTG
188	ACGTCAAAGGGCGATAAAGCACTAAATCCACACCC
189	TCTATCAGGGCGATTTTTTTGGGGTCGAGCCGCTA
190	TGAGTAACAGTGCCGAGG

Table S2. Nucleotide sequences for the staple strands containing the binding sites for HG-XDH with CH modification [S2, S3]

**Oligo DNA**	**Sequence (from 5' to 3')**
Binding site 1	TACCAGATTAAGACGT^{CH}TCATGAGTCATGAGTTTTCT^{CH}CATGACTCATGAACTCCTTATCGATTGA
Binding site 2	TGTAAATGATAGCTGT^{CH}TCATGAGTCATGAGTTTTCT^{CH}CATGACTCATGAACTAGATTATTAATTA
Binding site 3	ATAACATGCAACAGGT^{CH}TCATGAGTCATGAGTTTTCT^{CH}CATGACTCATGAACGAAAAACGTAAGAA

CH modified amino-C6-T was denoted as T^{CH}.

Table S3. Nucleotide sequences of linker strands

Oligo DNA	Sequence (from 5' to 3')
Linker strand 1	AGAGTAGGATGAATCAATCGGCCAGTTTGGAACAAGAGCTTGA
Linker strand 2	ACGGTGACGAGAGGCCCTGTTTTATTAAAGAACGTGTAAAGGG
Linker strand 3	CGTAGCAAGGAACCTACAGTTAATGCCCCCGCC
Linker strand 4	GCTTAATGACTCCTCGTTTTAACGGGGTCCTTG
Linker strand 5	AAGCCAACCCAGAATTGATGATGGGGTTTTGCTCAG
Linker strand 6	TTTAACAAGGTGCCGAAAACCGGCGGTCCACGCTGGGGTGGTT

Table S4. Nucleotide sequences of staple strands modified with Cy3 (5' modification) or Cy5 (5' modification)

Oligo DNA	Sequence (from 5' to 3')
Cy3-modification	**Cy3**-ATTAAGACACCCTCCCCATGTTTTG
Cy5-modification	**Cy5**-TGAGTAACAGTGCCGAGG

Table S5. Average number of assembled enzyme on DNA scaffold

Scaffolded enzyme	Modular adaptor enzymes	Number of well-formed DNA scaffold	Numbers of enzymes on modified sites [yield]				Average number of assembled
			Three-binding	Two-binding	One-binding	Empty	
**HPO/XDH**	HG-XDH	250	189 [75.6%]	21 [8.4%]	24 [9.6%]	16 [6.4%]	2.53[1]

Note:

[1] Calculation of average assembly yield of HG-XDH on DNA scaffold

$N_{\text{HG-XDH}} = 3 \times 75.6\% + 2 \times 8.4\% + 1 \times 9.6\% + 0 \times 6.4\% = 2.53$ (molecules of HG-XDH dimer on each DNA scaffold)

Supplementary references

S1 Douglas, S. M., Marblestone, A. H., Teerapittayanon, S., Vazquez, A., Church, G. M., and Shih, W. M. (2009). Rapid prototyping of 3D DNA-origami shapes with caDNAno. *Nucleic Acids Res*. 37, 5001-5006.

S2 Nguyen, T. M., Nakata, E., Saimura, M., Dinh, H., and Morii, T. (2017). Design of modular protein tags for orthogonal covalent bond formation at specific DNA sequences. *J. Am. Chem. Soc.* 139, 8487-8496. doi: 10.1021/jacs.7b01640

S3 Nguyen, T. M., Nakata, E., Zhang, Z., Saimura, M., Dinh, H., and Morii, T. (2019). Rational design of a DNA sequence-specific modular protein tag by tuning the alkylation kinetics. *Chem. Sci.* 10, 9315-9325. doi: 10.1039/C9SC02990G
